# Paleopathological Considerations on Malaria Infection in Korea before the 20th Century

**DOI:** 10.1155/2018/8516785

**Published:** 2018-05-09

**Authors:** Dong Hoon Shin, Min Seo, Jong Ha Hong, Eunju Lee

**Affiliations:** ^1^Lab of Bioanthropology, Paleopathology and History of Diseases, Department of Anatomy/Institute of Forensic Science, Seoul National University College of Medicine, Seoul, Republic of Korea; ^2^Department of Parasitology, Dankook University, Cheonan, Republic of Korea; ^3^Department of Internal Medicine, Asan Medical Center, University of Ulsan College of Medicine, Seoul, Republic of Korea

## Abstract

Malaria, one of the deadliest diseases in human history, still infects many people worldwide. Among the species of the genus* Plasmodium*,* P. vivax* is commonly found in temperate-zone countries including South Korea. In this article, we first review the history of malarial infection in Korea by means of studies on Joseon documents and the related scientific data on the evolutionary history of* P. vivax* in Asia. According to the historical records, malarial infection was not unusual in pre-20th-century Korean society. We also found that certain behaviors of the Joseon people might have affected the host-vector-pathogen relationship, which could explain why malarial infection prevalence was so high in Korea at that time. In our review of genetic studies on* P. vivax*, we identified substantial geographic differentiation among continents and even between neighboring countries. Based on these, we were able to formulate a strategy for future analysis of ancient* Plasmodium* strains in Korea.

## 1. Introduction

Globally, malaria is the fifth deadliest disease, infecting approximately 200 million people worldwide [1–3]. Malarial infection is mediated by the arthropod vector* Anopheles* mosquito. The* Plasmodium *parasite has a complex life-cycle of sexual reproduction inside the mosquito vector and asexual stage in the vertebrate host. In brief, malarial sporozoites are inoculated into human hosts when a mosquito bites them [4]. After a dormant phase, they differentiate into merozoites for release into the bloodstream, upon which they invade erythrocytes (the beginning of asexual multiplication). The bursting of infected red blood cells (RBCs) by merozoite multiplication is responsible for the typical malarial fever [4]. Some of the merozoites then develop into gametocytes, which are taken up by a female mosquito [4]. Sexual reproduction in the anopheline mosquito is followed by sporozoite migration into the salivary gland, from which they are inoculated into a vertebral host, thus beginning a new cycle of malarial infection [4].

In general, five species of genus* Plasmodium *are known to cause malaria:* P. falciparum, P. vivax, P. malariae, P. ovale, *and* P. knowlesi *[5]. Recent malaria outbreak in Brazil has also been traced to new zoonotic transmission of* P. simium* from monkey [6]. Among them,* P. vivax *and* P. falciparum* are the most commonly detected causative pathogens of human malaria. Clinical manifestations of uncomplicated malaria are nonspecific: headache, fever, malaise, myalgia, nausea, vomiting, and abdominal pain. Rare cases of malaria show severe manifestations including anemia, thrombocytopenia, pulmonary edema, renal failure, hepatic dysfunction, and splenic rupture [7].

Although malarial species share typical signs and symptoms such as undulating intermittent fever, they also have different traits depending on each subtype.* P. falciparum*'s symptoms are serious enough to show the highest mortality rate whereas* P. vivax, P. malariae,* and* P. ovale* exhibit generally nonfatal clinical courses [5]. The geographical distribution of each* Plasmodia *subtype differs as well.* P. falciparum* is more prevalent in tropical or subtropical zones including Sub-Saharan areas but relatively absent in temperate countries [8]. Meanwhile,* P. vivax* generally infects human populations in temperate and tropical zones but is not so prevalent in Sub-Saharan Africa [1, 3, 9].* P. vivax *malaria was endemic even in some high latitude countries (Finland and Russia, etc.) at certain points in history [4, 10, 11].

As malaria historically has been, and continues to be, one of the most serious diseases, it has attracted the attention of many paleopathologists. Studies on ancient malarial infection have been conducted by various methods such as osteoarchaeological and biomedical approaches [12]. As chronic-stage* vivax* malaria was generally known to induce anemia, further causing porotic hyperostosis (PO) or cribra orbitalia (CO) in the cranium [10], anthropologists have searched for the presence of PO or CO in skeletal remains as indirect evidences of malarial infection [5, 12–15]. Nevertheless, PO or CO has clear limitations with respect to its application to the study of ancient malaria because* Plasmodium* infection is not the only cause of them [12, 16]. Other pathologies such as inherited hemolytic anemia, scurvy, or malignancies are also known to induce the same skeletal changes of PO or CO [5, 12].

In recent years, the paleopathological study of malaria has been revolutionized by successful applications of immunological and ancient DNA (aDNA) analyses to archaeological specimens. To detect malaria-related proteins, researchers performed the dipstick assay or new-generation immunoassays on ancient mummies[17–19] or skeletons [20, 21]. The immunological assay became an effective screening method to secure the evidence of ancient malarial infection [5]. Also,* Plasmodium *aDNAs reportedly have been obtained from Egyptian mummies [2, 22–24], an infant skeleton dating to ancient Rome [25], 15th-to-19th-century infant of Bavaria [26], and 1st-to-2nd-century adult skeletons of Italy [27]. As a paleopathological tool, aDNA analysis is useful for confirming the presence of malarial genomes remnant in archaeological specimens as well as for revealing the origin and dispersal of the protozoan parasite in evolutionary history [28].

Although immunological and molecular analyses have become more reliable tools for the study of ancient malaria, the data obtained to date are not sufficient in terms of quantity and quality [16]. Moreover, since previous studies have focused mainly on ancient Egyptian, Roman, and Renaissance European remains thus far [5], such information as has been obtained from malaria aDNA reflects a serious geographical bias. Extensive geographic sampling is thus necessary in order to understand the demographic history of malaria much more comprehensively and clearly [4, 29–31]. Like the other continents, Asia is also a region where malaria has historically been epidemic and endemic. In several Asian countries, many people continue to have suffered from and even died of* Plasmodium* infection. Nonetheless, most of the requisite paleopathology still remains to be revealed in Asia as few medical studies on the ancient malaria have been reported in the area. Herein, then, we offer this historical review as a fundamental basis for future research of ancient malaria infection in Korea and other Asian countries.

## 2. Origin and Dispersal of Vivax Malaria Parasite

Parasitologists have speculated that human malaria might have been transmitted from nonhuman primates by a host-switch event [11]. Initially, they presumed that* P. falciparum* was transmitted from chimpanzees and gorillas in Africa [18] while* P. vivax* originated from another nonhuman primate, possibly macaques, in Southeast Asia [4, 32, 33]. However, this hypothesis is seriously challenged nowadays by the genetic analysis of malaria worldwide. Alternatively, a recent study revealed that both* P. falciparum* and* P. vivax* originated in Africa and that* P. vivax* transmission to human beings might have occurred much earlier than* P. falciparum *did by a host switch [34].

According to the estimated time to most recent common ancestor (TMRCA), the ancestor of the extant* P. vivax* populations existed between 50 and 550 ka before the present [3]. In a demographic history inferred from the* P. vivax* genome analysis, the global population size of vivax malaria might have expanded slowly until about 60 ka BP, which is closely consistent with the demographic history of mankind [3, 35]. Once the divergence of African and Eurasian* P. vivax* populations occurred at about 51 ka BP, the latter appears to have undergone a rapid exponential increase in population size [3, 35]. Among Eurasian vivax malarias, the East Asian variety might have experienced a distinct pattern of population growth [3]. The population of East Asian variety might have been relatively stable in its expansion until approximately 10,000 years BP [36, 37]. It then began to increase rapidly once rice and millet started to be domesticated in the area and sustained such increase, without tapering off, until the present [3]. The inferred hypothesis is suggestive of the detailed evolutionary history of vivax malaria in East Asia [3].

In the phylogenetic tree of* P. vivax *worldwide, two divergent groups were identified: a large star-like cluster and a divergent cluster [3]. The latter was composed of two subclades with different geographical distributions:* “Asia a”* of Central China and* “Asia b”* of China, Korea, and Indonesia. The divergent East Asian* P. vivax* lineage was connected to the large star-like cluster by a group of haplotypes found in Southeast Asia [3]. Based on the phylogenetic analysis, East Asian* P. vivax* might have been split from all other vivax malaria and developed a distinct demographic history for at least 121 ka [3]. Meanwhile, mutations of* P. vivax*-resistant RBCs (Duffy-negative phenotype) occurred in Sub-Saharan peoples [38]. Due to the mutations, vivax malaria disappeared completely from the area until the reintroduction of* P. vivax* to East Africa by sea-going traders from Asia [39].

## 3. History of Malaria Infection in Korea

The historical record is important for understanding the pattern of malarial infection in ancient civilizations[27]. In the classical period of Greece, Hippocrates famously described the typical undulating fever, a very suggestive sign of malarial infection [5, 39]. Historians believed that malaria became hyperendemic in Europe by its spread around the Mediterranean area, next along the riverbanks of the Rhine, Danube, and Rhone and then further to Northern Europe, while accommodating to colder climatic conditions in those areas [40]. Historical studies have shown that malaria became remarkably prevalent in the marshy areas of Northern Europe in the Early Middle Ages [5, 40, 41]. By the Later Middle to Early Modern Ages, except for Iceland, plenty of reports on malaria were available from every corner of Europe (including the North Sea, Germany, Anglo-Saxon England, and even Scandinavian countries) [40, 42, 43]. In a sense, malaria appears to have been a much more serious disease than even the Plague [40, 44].

Malaria must have been endemic in East Asia from ancient times as well, as descriptions about malaria-like symptoms can be seen in Chinese historical records [45]. Although Korea had been in close interaction with China from earliest times, in Korean history, the first recorded case of malaria occurred only in the Goryeo Dynasty (918-1392 CE) [46]. In a 14th-century record, a Joseon King's mother (Joseon Dynasty: 1392-1910 CE) was seriously infected with malaria and eventually died of it [47]. Over the following centuries, a wealth of records on the typical signs and symptoms of malarial infection (intermittent fever, repeated every third day) can be found in the Korean historical literature [47]. As most malarial infection in modern Korea has been revealed to have been caused by* P. vivax* [48], the Joseon people might have suffered from the same* Plasmodium *subtype. Notwithstanding the benign traits of* P. vivax*, relapsed infection typically might have exhausted people, often eventually killing them, as seen in similar clinical reports today [3, 49–53].

Before the first modern medical record on malaria in Korea (1886), prevalences of malarial infection could not be reliably calculated. In the* First Annual Report of the Korean Government Hospital, Seoul*, Dr. Horace Newton Allen described “endemic intermittent fever” (possibly malaria) as the most commonly observed sign among Korean patients who visited his hospital [54, 55]. According to him, in the late 19th century, hyperendemic malaria posed a serious threat to Koreans throughout the entire Joseon Kingdom. How, exactly, did malaria show such a high infection prevalence in Joseon society? In general, wetlands such as scattered swamps, bogs, and river valleys have been important habitats for anopheline mosquito breeding. As wetlands were distributed widely in Korea at that time, they must have been integral to the high malarial transmission rates [34, 40, 56–63].

In malariology, however, the waxing and waning of malarial infection in a specific area cannot be explained so simply. In addition to wetlands, environmental alteration or degradation due to human activity also has a great influence on the density and activity of mosquito populations and, further, on the prevalence of malaria itself[64–66].  Table 1 summarizes the anthropogenic causes of malaria currently recognized by scholars. As is apparent, people's efforts to exploit environments often induce outbreaks of malaria [40, 60, 64, 67]. Indeed, agricultural development and malaria are highly correlated in human history [60, 68–72]. The expansion of irrigation facilities, the reclamation of wetlands, economic specialization in agriculture, the simplification of crop types, enlargements of rice paddies, high population densities, deforestation, and still other malaria-inducing factors have been commonly cited (Table 1).

We do not yet know whether the close relationship between environmental change by agriculture and malarial infection is a universal phenomenon beyond certain temporal and spatial limits. In a recent cross-national analysis, however, correlations among anthropogenic activity, mosquito population sizes, and malaria rates were seen to have been common in many parts of the world [64]. The findings of Table 1 can thus be applied to our conjecture about Joseon society's vulnerability to malarial infection. In our careful examination of the Joseon records, we found many similar malarial-infection-facilitating situations to those noted in Table 1. The situations in Joseon society are summarized in Table 2.

In brief, the 15th to 19th centuries in Korean history were a turbulent and dynamic period during which the Joseon people were highly motivated to be involved in agricultural innovation, thereby eventually effecting major changes in their sociocultural environment (Table 2). By infusion of labor and capital investments into land development, the state of the agricultural techniques was advanced. By clearing every corner of wasteland and reclaiming wetlands, huge areas of farmland in the Kingdom were newly opened up [73–77]. Farmers cleared slash-and-burn fields even up to the tops of mountains[63, 73, 76]. By the end of the Joseon Dynasty, there was virtually no land remaining that had not been utilized for farming purposes (Table 2).

On such lands, Joseon farmers planted crops. Rice was very popular, becoming the most preferred crop by the late Joseon Dynasty [73]. To meet growing market demand for rice, farmers hastily turned their existing dry fields into rice paddies [54, 63, 73–75, 78, 79]. To supply enough water for rice cultivation, irrigation systems comprised of reservoirs and dammed pools were newly constructed in the Kingdom [54, 63, 74, 75, 78, 80]. Due to such increased agricultural productivity during the 15th to 19th centuries, the population of the Joseon Kingdom soared [73, 76]. All of these changes meant that the Joseon people came to live more and more in highly populated villages, towns, and cities around which rice paddies, reservoirs, and dams were scattered (Table 2). Certainly, as long as this new situation continued, malarial prevalence was by no means lowered. In a sense, intensive farming appears to have been a necessary evil for the Joseon people, as, notwithstanding the malaria-inductive environments thus created, the increased food production potentiated and achieved thereby was a great economic as well as social boon to the Kingdom.

From the late 19th century, the diagnosis and treatment of malaria began to be performed by specialists in Western medicine. In 1913, for example, an intermittent fever observed among Korean patients was finally confirmed by a modern microbiology technique to be* Plasmodium *infection [54, 81]. During the Japanese colonial period, however, significant reduction of malarial incidence proved difficult, as the environmental conditions associated with agriculture remained the same. Since the end of World War II and subsequent US army administration, malarial infection as well as its management underwent a revolutionary change in Korea. The US army, which had experienced many deaths from malaria in the course of the war with Japan, was able to establish an effective means of controlling the* Plasmodium *infection in Asia [79]. Whereas the Japanese during the period of their colonial rule of Korea preferred a strategy entailing curing of malaria patients by quinine treatment at the onset of the disease, the US military administration adopted a far more aggressive and highly effective policy of controlling malaria through the use of insecticides [79].

However, we must also consider the possibility that malarial prevalence in the country was not reduced by antimalarial medications or insecticides alone. This idea is supported by instances in European history. In the 18th to 19th centuries, malaria was still prevalent in Europe, though it rapidly declined thereafter, finally disappearing from most regions by the 1930s [82]. The retreat of malaria from Europe was not the result of medical or chemical innovations, as no such deliberate countermeasures (entailing use of quinine or insecticide, etc.) had been pursued at that time [83]. Rather, another factor has been proposed to explain the decline of malaria in Europe: socioeconomic progress [84]. Many studies in fact have shown a positive correlation between malaria and poverty [85, 86]. Furthermore, it has been established that malaria was active for much longer periods of time in regions where modernization was delayed [40]. In Finland, long-term social changes such as land consolidation, decreasing household size, fewer interactions between families, and the transition from extended family to nuclear family have been posited as causes of malaria's decline during the past 200 years [83]. In fact, the combined effects of social innovations and improved standards of living were decisive in controlling and eventually eradicating malaria in Europe [40, 60]. Arguably, this explanation is applicable to the decline of malaria in South Korea as well. In that country, malaria was eradicated in the 1970s by the cooperative efforts of the World Health Organization (WHO) and Korea's National Malaria Eradication Program [48, 87]. Significantly, this corresponded to the period of rapid industrialization by which the living standards of the Korean people were remarkably improved. In short, socioeconomic development in South Korea might have made a great contribution to the eradication of malaria in that country.

Unfortunately, the reemergence of malaria after long-term eradication is not a rare phenomenon in the world[3, 88]. In 1993, after more than two decades of malaria-free status, a new Korean malaria patient was reported among soldiers who had served near the Demilitarized Zone (DMZ) in South Korea [48, 89]. At the time, as North Korea was suffering malarial infection, the new patient was thought to have been infected by an* Anopheles* mosquito migrating from the north [87, 90]. In fact, this cannot explain everything about the reemergence of malaria in South Korea, as, nowadays at least, foreign travelers and workers from malaria-endemic regions are commonplace [87]. Today, malaria is once again an endemic disease and a source of public concern in South Korea, as cases of malarial infection have continued to be reported [87, 90, 91].

## 4. Genetic Diversity of Modern Korean* P. vivax* Isolates

Malarial infection in history often cannot be fully evidenced by the examination of historical documents. This is due to difficulty in accurate diagnosis of ancient malaria cases in history. Actually, the signs and symptoms of ancient malaria patients were often vaguely described in historical literatures by modern clinical medicine standards. In addition, diagnosis of ancient malaria (solely) by the examination of archaeologically obtained skeletal remains is also highly problematic as malaria leaves little traces on bones. In this regard, we note that DNA-based study could be useful for acquiring scientific evidences of specific diseases prevalent in history[27].

DNA analysis of* P. vivax* is generally targeted on the protozoan parasites' surface proteins by which the erythrocyte invasion of the* vivax*-malarial parasite can be triggered [92, 93]. One such surface protein is the* P. vivax *merozoite surface protein (PvMSP), which is abundantly expressed on the merozoites of vivax malaria [94]. Duffy-binding protein (PvDBP) is another membrane protein that is also present on the* P. vivax* merozoites and that plays a crucial role in RBC invasion of parasites [92, 93, 95, 96]. As antibodies against these proteins effectively block the invasion of* P. vivax* into human RBC[91], PvMSP and PvDBP are regarded as leading candidates for use in the development of malaria vaccines[91, 97–99] though great genetic diversity among those surface proteins still represent a major obstacle to the vaccine research [4, 91, 97, 100–103].

Since the reemergence of* P. vivax* in South Korea [48, 87], Korean researchers also have aimed to study the genetic traits of vivax malaria's PvMSP, PvDBP, circumsprozoite protein (PvCSP), apical membrane antigen-1 (AMA-1), microsatellites sequences, and 18S ribosomal RNA genes [87, 90, 91, 103–108]. Those scientists are indeed eager to analyze the genetic diversity, population structure, and operation of natural selection among Korean* P. vivax* isolates, as the outcomes would doubtlessly be useful for understanding the nature of the* P. vivax* population in South Korea [90, 91, 97]. In general, the genetic diversity of* P. vivax* is higher than that of* P. falciparum*, suggesting that the former has a long, complex, but successful evolutionary history of adaptation [4, 109–112]. However, when* P. vivax* reemerged in South Korea, the isolates of the years 1993 to 2000 were genetically closely related, meaning that its genetic diversity was very low at the initial stage of its reintroduction [113]. Since 2001, the reemergent malaria population in South Korea has become more heterogeneous, showing increased genetic diversity and a more complex population structure [87, 89, 97, 108]. The results clearly indicate that some genotypes that were not found before 2000 eventually migrated into South Korea at a much later date, as accompanied by outbreeding between different genotypes [90].

**Figure 1:**
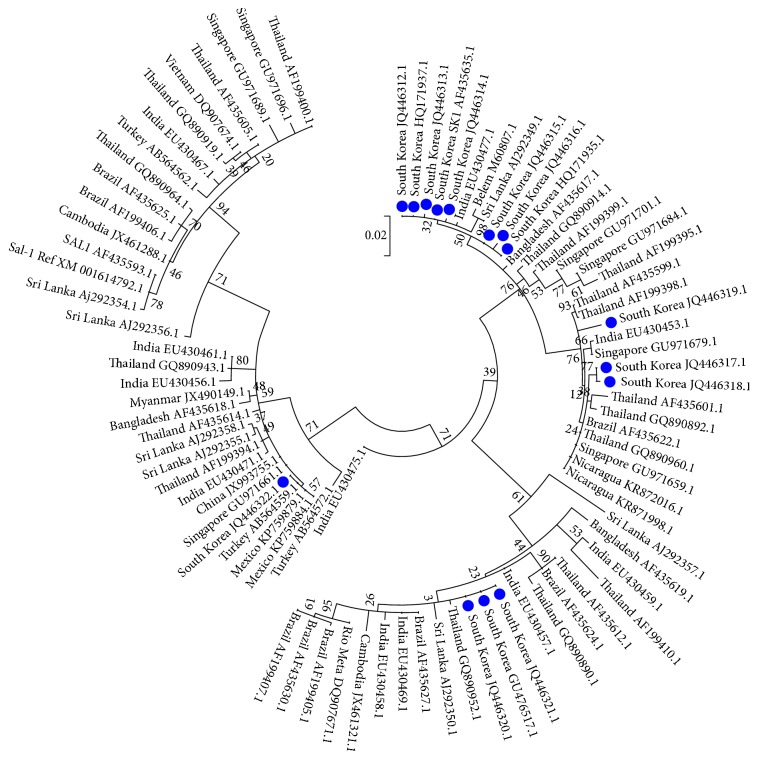
Maximum Likelihood (ML) tree of* Plasmodium vivax *MSP-1_42_ gene sequences reflecting the genetic traits of South Korean isolates (blue dots) since the reemergence of* P. vivax* in 1993. We inferred the ML tree by MEGA6 program [114], based on the research result of Kang et al. [97]. Tree building also used additional MSP-1_42_ gene sequences (*n* = 18) collected from GenBank: South Korea (GU476517.1; HQ171935.1; HQ171937.1), Bangladesh (AF435619.1), Brazil (AF435625.1; AF435627.1; AF435630.1), Cambodia (JX461288.1; JX461321.1), China (JX993755.1), Mexico (KP759879.1; KP759884.1), Myanmar (JX490149.1), Nicaragua (KR871998.1; KR872016.1), Rio Meta (DQ907671.1), and Thailand (AF435599.1; AF435605.1). Bootstrap values were made for ML tree [115]; the number in the branches indicates bootstrap proportions (1,000 replicates). Scale is in substitutions per variable site. Support values were calculated using Hasegawa-Kishino-Yano model. In this tree, MSP-1_42_ taxa (*n* = 86) could be classified into the five separate clades. South Korean* P. vivax *taxa (*n* = 15) belong to four different clades among them. Half of them were essentially similar to the Belem type; the others were recombinant forms between Sal-1 and Belem.

Ju et al. [91] also reported that a phylogenetic analysis based on PvDBPII sequences showed 3 different clusters (SK-1, SK-2, and SK-3) in Korean* P. vivax* isolates. Among them, SK-3 was a new clade that had not been identified at the early phase of reemergence in the same Korean isolates [106] but later became a more prevalent group than either SK-1 or SK-2 [91]. They agreed that the polymorphic nature of the PvDBPII of recent malarial isolates is distinct from those isolated at the early phase of malaria's reemergence in South Korea. The value in the rate of nonsynonymous and synonymous mutations (dN-dS) also implied that PvMSP in Korean* P. vivax* isolates has been under the strong influence of positive natural selection [97] (Figure 1).

## 5. Paleopathological Approach to Ancient Malaria Infection in Korea

The recent studies on genetic diversity, gene flow, and population structure are also very significant to the development of strategic control measures against vivax malaria after its reemergence in South Korea [90, 112, 116]. Despite all the benefits, however, the overall genetic trends of vivax malaria, especially concerning its evolutionary history, have not yet been revealed by the simple genomic assay of modern isolates. In fact, the investigation of* P. vivax* using present-day DNA extracts from modern Korean isolates often leads to confusion as to vivax malaria's origin and dispersal [28]. To overcome this drawback, we must conduct aDNA analyses on various human samples obtained from archaeological sites in Korea to analyze the genetic origin and phylogenetic history of malaria more accurately and comprehensively.

The significance of aDNA analysis to any derived understanding of the evolutionary history of malaria recently has been demonstrated by Gelabert et al. [28]. By way of aDNA analysis on 70-year-old microscopic slides of blood from malaria-infected people in Spain, they were able to successfully reconstruct the mtDNA sequence of the now-eradicated European* P. vivax* malaria. Moreover, as it was proven to be related to the most common present-day American* P. vivax* haplotype, the authors were able to confirm that vivax malaria entered the Americas by post-Columbian contact with Europeans [28]. In this way, aDNA assay of ancient human remains can be used for finding the missing links in the origin and spread of ancient malaria.

In aDNA analysis, the types of specimens to choose are very crucial to the research's success. To select the specimens ideal for aDNA assay purposes, the life-cycle of the vivax-malarial parasite must be considered. In brief, when malarial sporozoites are inoculated into human hosts, some of them migrate to the liver wherein they invade the hepatic parenchymal cells [4]. While some sporozoites can maintain the dormant state there, they can be further differentiated into merozoites and released into the bloodstream [4]. As seen in vivax malaria's life-cycle, the liver is the place where the final preerythrocytic phase takes place [45]. In this regard, Joseon Dynasty (1392-1910 CE) mummy's livers might be significant to our project. For the past 10 years, scientists and archaeologists in South Korea have been involved in interdisciplinary work on well-preserved mummies discovered in Joseon Dynasty tombs [117–123]. The livers that could be used for aDNA analysis were obtained from mummies by en bloc resection during autopsy (Table 3) [117].

Nevertheless, as the number of malaria sporozoites at liver stage might be actually very small, we should also consider alternate specimens for our aDNA analysis. In this regard, we note that a small amount (less than 1 g) of spongy bones inside vertebrae (possibly containing hemopoietic cell remains) was chosen commonly as specimens for aDNA analysis of malaria; and in another case, first or second molars have been selected for* Plasmodium* aDNA analysis [27]. Many future studies on ancient malarial genomes will proceed with these specimens of Korean mummies or skeletons.

## 6. Conclusion

With respect to human samples obtained from archaeological sites, scientific techniques can be done to reveal whether the individual had suffered from malaria in his lifetime or to obtain phylogenetic information of its ancient genome. As the previous studies on ancient malaria have focused mainly on specimens from Egypt and Europe, however, the current information so far obtained carries a serious geographical bias. More extensive geographic samplings and assays are thus needed in order to obtain a more comprehensive demographic evolutionary history of malaria.

Like the other continents, Asia is a region wherein malarial infection has been epidemic in history. Nonetheless, very little has been done in the way of relevant paleopathological studies on ancient malaria. We thus reviewed the history of malaria in Korea and attempted to derive scientific clues to the evolution of* P. vivax* there and elsewhere in Asia. To those ends, we first examined the historical-documentary evidence of ancient malarial outbreaks in Joseon society and found that malarial epidemics were in fact not unusual in pre-20th-century Korea. We detected changes in the host-vector-pathogen relationship, which probably affected the proliferation of the mosquito vector and indeed the prevalence of ancient malaria in Joseon society. We also noted, in our review of genomic studies on* P. vivax*, substantial geographic differentiation of vivax-malarial DNA between different continents and even neighboring countries. Many scientific studies on the history of malaria will be done with ancient specimens in Korea and Asia, pending the permission of the relevant medical-ethics review boards.

## Figures and Tables

**Table 1:** The factors proven to relate with high prevalence of malaria infection.

Factors	Details	References
Marsh, wetland, coastal area, swamp, etc. as the possible source of malaria infection	In the African countries, anopheline larvae were abundantly found in the swamps; and topographic wetness (of wetlands) was strongly associated with the spatial distribution of malaria infection cases. The control of wetlands is important for malaria elimination.	[34, 40, 56–62]
	In the late Bronze to early Iron Age Europe, the occupation of the coastal marshes paved the way for the spreading of malaria. Mosquito larvae were able to grow up in stagnant pools and ditches of North Sea or Anglo-Saxon England coast marshes. In European history, the coastal marshes were generally hyperendemic for malaria during 16th to 18th century.	

Expansion of irrigation facilities	In Ancient Egypt and Rome, Fayum area became the granary by repeated projects of large-scale land reclamation and construction of canal system. *Anopheles* vector breeding was directly or indirectly linked to the presence of extensive irrigation system. Fayum was seriously exposed to the hazards of malaria by increasing contact between human beings and mosquitos that were bred in the newly developed irrigation system.	[2, 60]

Reclamation of wetland for agriculture	The linkage of land reclamation and malaria infection was proven in East Africa. For instance, the elimination of papyrus from the wetlands during land reclamation promotes the breeding of mosquitos and malaria infection. Drainage ditches in newly claimed agricultural land were also the most common breeding site for mosquitos. In brief, the land reclamation was to foster mosquito reproduction by reduced vegetation cover and the elevation of the temperature at breeding site.	[2, 60, 124, 125]

Intensified crop cultivation or economic specialization in agriculture	Epidemiological study showed that malaria transmission and intensified crops are highly related. The incidence of malaria is about ten times higher in cereals-cultivation area than in areas with less cereals. Intensity of crop cultivation is highly associated with exacerbated human risk of malaria. Specialization in agriculture initially influences on forest loss, and further induces malaria infection (by reducing the biodiversity due to a replacement of huge variety of vegetation with nonnative crops).	[2, 64, 124–127]

Rice cultivation	The rice paddies provide abundant breeding opportunities for malaria mosquitos. It also becomes a challenging site for vector control. Improper drainage from rice paddies caused Anopheline mosquitos to breed. Mosquito control agents are difficult to be applied to rice paddies. Rice cultivation has a deep-rooted relationship with malaria transmission.	[124, 128–133]

Deforestation	The pupation rate of *Anopheles* mosquito was the highest in the samples collected from deforested areas. Land cover pattern is a key factor that influences the habitat for malaria mosquitos. Relationship between deforestation caused by a small-scale farming and *Anopheles *mosquito breeding was evidently proven in Amazon region.	[60, 64, 65, 134–144]
	In a structural equation model across 67 (developing) nations, positive association was observed between deforestation rates and malaria prevalence. In Sub-Saharan countries, living in the land without trees led to the increased risk of malaria infection. The relationship between land cover and the reproduction of malaria vector mosquitos was also shown in Western Kenyan Highlands.	
	Epidemiological aspects of ecosystem change (deforestation) and mosquito habitat proliferation (increased levels of larvae, mosquito populations, and actual malaria rates) have been studied extensively. Significant relationship was observed between the percentage of forest cover loss and higher infection prevalence of malaria.	
	Deforestation impacts malaria prevalence by multiple mechanisms: increase in the sunlight amount, warming temperature ideal for the pupation of malaria vector larvae, standing water after clearing terrain, the land becoming flatter and more likely to store water, which is typically less acidic and more conductive to *Anopheles *larvae development.	
	When the forest is replaced by new croplands, the plants still provide the bushy cover for mosquito proliferation, making the malaria infection prevalence higher.	

High population density	In developing countries, rural population growth and needs to increase food production induce the forest loss, further influencing malaria infection. Using the timbers for building and fuel wood is also one of the key causes of deforestation. Growing rural population also tend to live closer to the natural habitats of mosquitos, further experiencing risk of malaria infection.	[40, 64, 145–147]
	In medieval city of Groningen, 10 percent of the urban population died while the surrounding countryside showed a death rate of 5 percent.	

**Table 2:** Historical findings possibly related to the malaria outbreaks in Joseon society.

Changes in history	Date	Historical details	References
Increase in the ratio of rice paddies to total cultivated land	15th to 18th century	Piling up reservoirs or dams to turn wetlands into rice paddy fields. At the late 17th to 18th century, rice cultivation became dominant in the agriculture of Joseon Dynasty.	[63, 73]
	18th to 19th century	Changing cultivated dry fields to the rice paddies (42.9% in 1759 to 68.6% in 1901). One-third of rice paddies of Joseon Kingdom was created at this period.	[73–75, 78, 79]
	Japanese colonial period	Changing the dry field to rice paddies still continued in colonial Korea. *Anopheles sinensis*, the vector mosquito of malaria in Korea, usually propagates in the stagnant water of rice paddies. Malaria infection was thus very common in the places.	[54, 79]
	US military administration	Trying to get rid of such mosquito propagation spots by making gutters to drain the water.	[79]

Construction of reservoirs or dams	15th to 19th century	From the late 15th century on, the irrigation system such as reservoirs and dams were actively built in Korea. The water in the dams could be drained into the paddies for rice cultivation. The construction and maintenance of irrigation system was governed by government or gentries in countryside (16th century). In the early 19th century, the number of reservoirs or dams reached as many as 5,960 in Korea. The construction of dams and reservoirs reached the peak in the early 19th century, maintained even in Japanese colonial period.	[54, 63, 74, 75, 78]

Simplification of crop types	Since 18th century	To benefits from higher productivity of rice farming and respond the market demands much efficiently, the proportion of rice in cereal cultivation has been greatly expanded.	[73]

Clearing and reclamation of the land; Slash and burn in mountain area	15th to 18th century	Large-scale reclamation was taking place in abandoned land, wetland, coastal, and low-lying areas at river or stream basins (15th to 17th century). Slash and burn increased in mountainous area (since 17th century). Previous ranch and isolated islands were also turned into farmland. The nonfarming backdrops almost disappeared throughout the country after mid-17th century.	[73–77]

Deforestation	Since 17th century	Population growth in Joseon society led to increased demand for forest products. Serious deforestation at this stage was also caused by slash-and-burn farming (after 17th century on). At this stage, approximately 40–50 percent of the cropland in Joseon Kingdom was prepared by slash-and-burn farming, causing deforestation in the mountain area. In mid-17th century, no matter how deep the mountain area was, there was no place where no cropland was. Deforestation induced frequent landslides and floods.	[63, 73, 76]
	Colonial period	Deforestation appears to have induced malaria in colonial period Korea.	[54]

Population increasing	Mid-16th century	9 to 10 million people in Korean peninsula.	[73]
	Late 18th century	Population in Korean peninsula reached 15 million. The increase in population at this stage appears to have been due to the high productivity of rice cultivation in Korean peninsula at that time.	[73, 76]

**Table 3:** Archaeological information on mummy samples with liver obtained during autopsy.

Number	Mummy	Estimated Date	Sex	Date of excavation (YYYY.MM)
1	Cheongdo	1642^a^	Male	2014.10.
2	Andong	18C^b^	Male	2013.01.
3	Dalsung	16C-17C^c^	Female	2014.05.
4	Hwasung	18C^c^	Male	2012.12.
5	Gangneung	1622^a^	Male	2007.11.
6	Hadong2	Late 16-early 17C^a^	Female	2009.06
7	Kunkook	Joseon period^c^	Female	Unknown
8	Mungyeong	1647^d^	Female	2010.04.

^a^Historical documentation. ^b^Carbon dating. ^c^Archaeological evidence. ^d^Tree ring.
